# Anticancer potential of natural bioactive compounds saponins in glioblastoma multiforme

**DOI:** 10.3389/fphar.2025.1712599

**Published:** 2025-11-13

**Authors:** Deena Elsori, Fahad M. Alshabrmi, Ahmed M. Alharbi, Mohd Saeed, Ajay Singh, Pratibha Pandey, Safia Obaidur Rab, Fahad Khan

**Affiliations:** 1 Faculty of Resilience, Rabdan Academy, Abu Dhabi, United Arab Emirates; 2 Department of Medical Laboratories, College of Applied Medical Sciences, Qassim University, Buraydah, Saudi Arabia; 3 Department of Medical Laboratory Science, College of Applied Medical Sciences, University of Ha’il, Hail, Saudi Arabia; 4 Department of Biology, College of Science, University of Ha’il, Hail, Saudi Arabia; 5 School of Applied and Life Sciences, Uttaranchal University, Dehradun, India; 6 Centre for Research Impact and Outcome, Chitkara University, Rajpura, Punjab, India; 7 Department of Clinical Laboratory Sciences, College of Applied Medical Sciences, King Khalid University, Abha, Saudi Arabia; 8 Center for Global Health Research, Saveetha Medical College and Hospital, Saveetha Institute of Medical and Technical Sciences, Chennai, Tamil Nadu, India

**Keywords:** saponins, natural compound, anticancer, apoptosis, glioblastoma multiforme

## Abstract

According to the WHO Organization, glioblastoma multiforme (GM) is classified as a grade IV histological malignant tumor of the central nervous system. Numerous genetic abnormalities (tumor cells) and dysregulated cell cycle checkpoints, such as G1/S, have been linked to the progression of glioblastoma multiforme. In addition to tumor removal, glioblastoma multiforme can be treated with chemotherapy and radiotherapy. Immunotherapy and medications that block integrin signaling pathways have also been used. Owing to tumor recurrence, metastasis, and treatment resistance, conventional pharmacological regimens continue to be lethal. Consequently, the quest for an efficacious therapeutic approach for GM remains elusive. Innovative products derived from natural substances have also been proposed as potential remedies. They safeguard glial cells by inducing apoptosis, reducing oxidative stress and neuroinflammation, suppressing proliferation, decreasing pro-oncogenic processes, and enhancing the effectiveness of anticancer therapies. Among various natural compounds, saponins are now being considered for use in cancer treatment. The function and mechanism of action of saponins in cancer have been reported in numerous research findings. These anticancer effects include antioxidant activity, apoptotic induction, cell cycle arrest, and suppression of cellular invasion and migration. This review presents the application of natural saponins as a potential alternative for developing a novel therapeutic strategy that will enhance the treatment options for combating GM in the future.

## 1 Introduction

**FIGURE 1:**
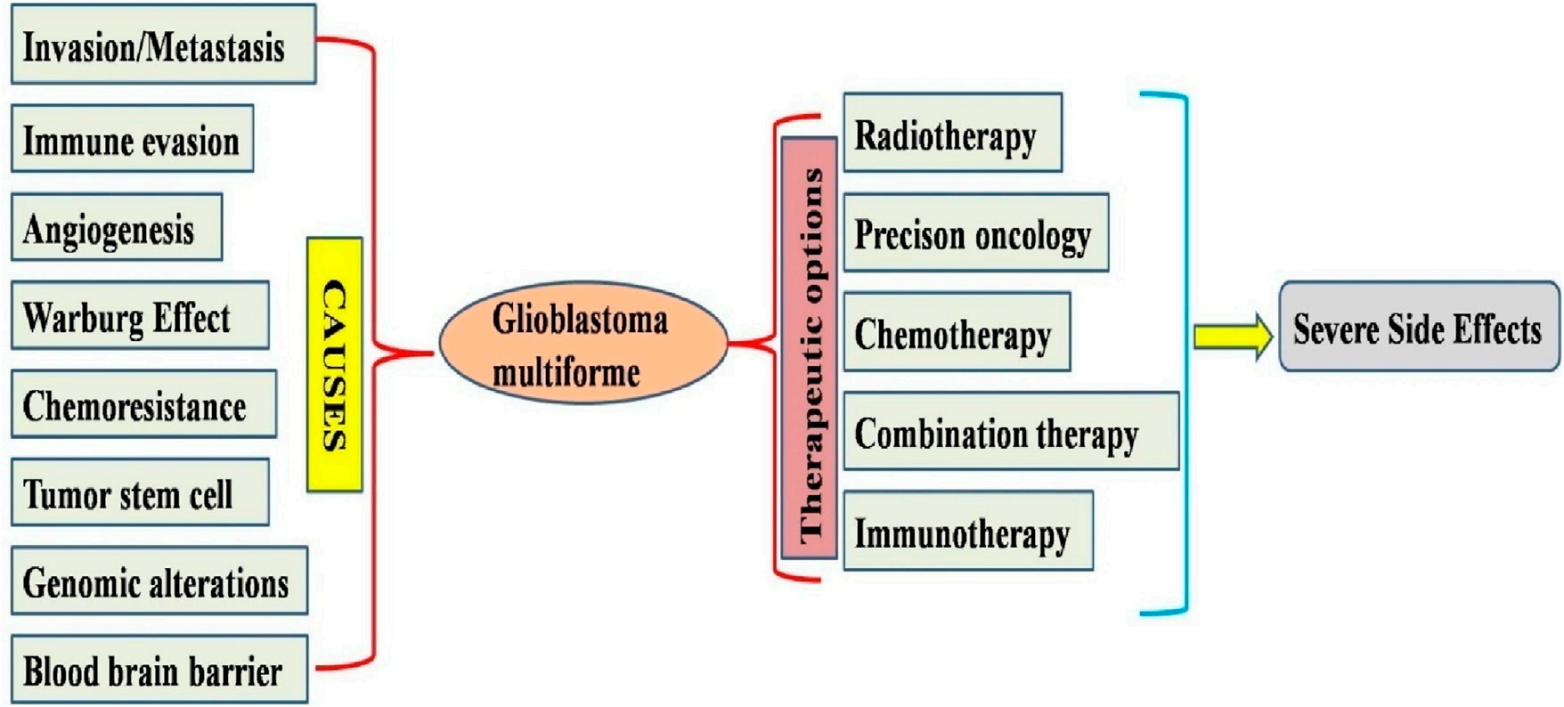
Diagram illustrating the mechanism behind the progression of glioblastoma and possible therapeutic options available for treatments available for glioblastoma multiforme.

Glioblastoma multiforme (GM) is a prevalent kind of malignant brain neoplasm in humans, constituting approximately 60% of all malignant brain tumors (Grochans et al., 2022b). According to the WHO classification, gliomas have four distinct forms: astrocytic tumor grades (I, II, III, and IV) (Perry and Wesseling, 2016). In the 2021 WHO classification, tumors are now graded within their specific tumor type, making terms like “anaplastic astrocytoma” obsolete. Under the 2021 WHO classification, a diagnosis of glioblastoma (CNS WHO grade 4) requires the tumor to be a diffuse astrocytic glioma that is IDH (isocitrate dehydrogenase) gene (without a mutation in the IDH1 or IDH2 genes) (Park et al., 2023). It is hard to diagnose GM because of its severely invasive nature and poor response to standard cytotoxic treatment. Unfortunately, GM is resistant to conventional therapeutic methods (Ou et al., 2020). Conventional therapies (chemotherapy and radiation) have caused significant adverse effects, including gastrointestinal complications, destruction of normal cells, multidrug resistance, and bone marrow suppression. Consequently, these research outcomes increased the demand for potent novel pharmaceuticals that can significantly reduce the adverse effects of conventional therapeutics used for GM treatment (Stylli, 2020) (Figure 1).

Excessive intracellular accumulation of ROS is considered a major trigger for apoptosis (Redza-Dutordoir and Averill-Bates, 2016). Recently, excessive ROS accumulation has been shown to result in the malfunctioning of mitochondrial membrane potential (MMP), lipid peroxidation, protein oxidation, oxidative DNA damage, and enzyme inactivation. Consequently, ROS are regarded as important targets for the development of anti-cancer pharmaceuticals. Antioxidant substances that enhance endogenous antioxidant defenses have been suggested for neurological protection (Korczowska-Łącka et al., 2023). Glioblastoma multiforme and other neuroinflammatory diseases like Alzheimer’s disease are biologically linked by common pathological mechanisms, including chronic inflammation, oxidative stress, and dysregulation of key molecular players like TP53, STAT3, AKT1, and IL6. However, the theme of this review article is about GM therefore it would be beneficial to focus on complex interplay at the genetic, molecular, and cellular levels, leading to similar disruptions in cellular signaling and the microenvironment. Identifying and modulating these shared molecular pathways using natural compound offers a compelling strategy for developing innovative therapies to treat or manage glioblastoma multiforme Pradeep et al., 2025. Phytotherapy is a highly effective method of overcoming resistance from the conventional cancer therapies such as chemotherapy and radiotherapy. Phytochemicals are categorized into many classes including alkaloids, saponins, phenols, organosulfur compounds, and terpenes (Piekarz et al., 2024).

Phytocompounds in these categories are derived or processed from specific plant sources. Saponins (natural glycosides) comprise triterpene or steroid aglycone, exhibiting various pharmacological effects, including notable anti-tumor action (Elekofehinti et al., 2021; Zhou et al., 2023). Saponins are known to interact with numerous proteins involved in molecular pathways related to GM progression and survival (Tian et al., 2013). Saponins have demonstrated significant immunomodulatory potential and are still under investigation for their efficacy in inhibiting immune checkpoints (Abbas et al., 2020). Consequently, more effective and potent reviews are essential to provide specific and detailed insights into the potential of saponins as anti-GM agents to formulate effective treatments. Therefore, we have presented all the potential saponins with antiGM malignancy which could aid in the saponin based therapies for the management of glioblastoma multiforme.

## 2 Natural compounds against glioblastoma multiforme

GM (Grade 4 astrocytoma (GM) is a highly aggressive and malignant (Liu et al., 2023). The conventional treatment protocol comprises surgery followed by concomitant radiation with temozolomide and subsequent adjuvant temozolomide therapy (Gupta et al., 2022). Despite extensive surgical excision and treatment, the overall prognosis of patients with GM remains unfavorable. The difficulties associated with GM primarily arise from the cell signaling and genetic pathways involved in the development of this tumor type (Grochans et al., 2022a). Genetic modifications in glioblastoma multiforme (GM) encompass EGFR amplification and CDK4 gene changes, deletion of cyclin-dependent kinase inhibitor genes, and silencing of the O-6-methylguanine-DNA methyltransferase (MGMT) gene (Yu et al., 2020). Several genetic modifications promote cancer cell proliferation, including angiogenesis, Akt/mTOR signaling, cell cycle progression, tumor metastasis, metabolic abnormalities, inflammation, and chemoresistance. Conventional oncological therapeutic seek to rectify the imbalance between apoptosis induction and cellular proliferation by suppressing cell growth and enhancing programmed cell death (Balca-Silva et al., 2019). These saponins upregulate apoptotic genes in several cell lines (Zhou et al., 2020; Mbaveng et al., 2020; Xu et al., 2020).

Consequently, the therapeutic efficacy of natural dietary compound in GM treatment was assessed. Diverse compounds present in vegetables, fruits, and other natural products promote GM apoptosis and inhibit tumor proliferation. Compounds from coumarins, alkaloids, carboxylic acid derivatives, steroids, curcuminoids, lignans, flavonoids, tannins, terpenes, terpenes, and carotenoids provide chemotherapeutic efficacy against GM. Consequently, they enhance the mortality of tumor cells by enhancing apoptosis and autophagic pathways while suppressing proliferative pathways (Zhai et al., 2021). Bioactive components of anticancer drugs include etoposide (from *Podophyllum peltatum*), vincristine (from *Catharanthus roseus*), camptothecin (from *Camptotheca acuminata*), and paclitaxel (from Taxus) (Tian et al., 2013).

Resistance to approved medications remains a concern, despite extensive research on GM therapeutics. Resistance to GM therapy has been extensively documented for numerous reasons, including faulty mismatch repair (MMR) proteins, clonal selection, cell developmental pathways, and MGMT repair enzyme function. Phytocompounds that address drug resistance are derived from specific plant sources through extraction, and can target many proteins by modulating molecular pathways linked to GM advancement and persistence. Phytocompounds have been utilized as immunomodulatory drugs but are under investigation for their use in the inhibition of immune checkpoints. Consequently, more significant innovative research is necessary to elucidate their efficacy against GM to produce effective treatments. Our review displayed detailed insight about the potential of one such phytocompound, saponin for the treatment of GM malignancy (Prakash and Gabrani, 2024).

## 3 Saponin: a phytochemical based therapeutic option for treatment of glioblastoma multiforme

Saponins are a class of natural glycoside compounds classified as triterpenoids glycosides or steroids glycosides. They differ in solubility and permeability characteristics and thus can be differentiated according to the biopharmaceutical classification system. Surfactants have the potential for drug delivery by dissolving cholesterol in the plasma membrane of cancer cells (Oakenfull and Sidhu, 2023). Certain saponins have demonstrated the ability to eradicate glioblastoma cells in numerous model systems, which gives them solid foundation for being developed as “stand-alone” medications or in the form of component of a drug combination (from the current drug arsenal) for glioblastoma treatment (Table 1).

**TABLE 1:** Overview of saponins with their sources and preclinical models targeting glioblastoma multiforme.

Type of saponin	Saponin origin	Preclinical research	Effective doses	References
Saponin B	*Anemone taipainsis*	U87MG cell	1.67–13.35 μmol/L	Wang et al. (2013)
Saponin 1	Rhizomes of *Anemone taipaiensis*	U251MG and U87MG glioma cell lines xenograft tumors derived from U251MG and U87MG cells of nude mice	7.4 μg/mL and 10 μg/mL through injection in tail veins	Li et al. (2013)
Acacic acid-type saponins (lebbeckosides A and B (1–2))	Roots of *Albizia lebbeck plant*	U-87 MG and TG1 cells derived from a patient tumor	3.46 μM and 1.36 μM for 1, and 2.10 μM and 2.24 μM for 2	Noté et al. (2019)
Madecassoside (MAD-encapsulated alginate-chitosan nanoparticles)	*Centella asiatica*	Madecassoside encapsulated alginate chitosan nanoparticles (MACNP)-treated C6 glioma cells	20–200 µg/mL	Kunjumon et al. (2021)
Ardipusilloside I (ADS-1)	*Ardisia pusilla*	U373 and T98G glioma cells	11.70 μg/mL, 9.09 μg/mL	Wang et al. (2014)
Ginsenoside Rh2 (triterpene saponin)	*Ginseng*	Human glioma cells U251 cells, transfection with an miR-128 inhibitor	12 μg/mL	Wu et al. (2011)
Platycodin D (PD)	*Platycodongrandiflorus*	U87MG, U373MG cells	10 μM	Lee et al. (2022b)
Raddeanin A (RA)	*Anemone raddeana Regel*	T98G, LN299, U87, U251, U87-luciferase, 6-week-old female BALB/c nude mice	100–800 nM and intraperitoneal injected with RA (100 mg/kg/day)	Wu et al. (2021)
Deapioplatycodin D (DPD)	*Platycodon grandiflorum*	U251 cells and mouse subcutaneous xenograft tumor model	5–80 μM and 4, 8 mg/kg body weight	Sun et al. (2025)
Dioscin	*Dioscoreaceae family*	C6 glioma cell, allograft rat model	1.25–5 μg/mL and 30 mg/kg dioscin orally	Lv et al. (2013)
Paris saponin H (PSH)	*Paris polyphylla*	U251 cells	25–100 μg/mL	Bi et al. (2021)
N45 (steroidal saponin)	Rhizome of *Paris vietnamensis*	U251, U87, and U87R (TMZ-resistant glioblastoma cells)	4 μg/mL	Cui et al. (2024) Zhang et al. (2020)
Diosgenin	Fenugreek and wild yam	Rat C6 and human T98G glioblastoma cells	5–25 µM	Khathayer and Ray (2020)
Timosaponin AIII (TIA)	*Anemarrhenaasphodeloides*	U87MG cells and tumor of U87MG cells implanted in nude mice	5–10 μM and 1 mg/kgI.P.	Liao et al. (2023)
		GBM8401, M059K cells and xenograft, orthotopic mice model	5–15 μM and 5, 10 mg/kg	Lee et al. (2022b)
Saikosaponin D	*Bupleurum falcatum*	U251, RG-2, and U87-MG cells	9–21 μM	Liu et al. (2022)
Saponin 6	*Anemone taipaiensi*s	U87 MG cells	2.83 and 5.66 µM	Ji et al. (2016)
Paris saponin VII	*Trillium tschonoskii Maxim*	U87-MG, U251, LN229, GL261 cells and GL261 xenograft mice model	0.25–1 μM and 2.5, 5 mg/kg I.P.	Wang et al. (2025)
Compound K	CK/ginsenoside metabolite	U373MG and U87MG cells	50 and 75 µM	Lee et al. (2017)
Spirostanol saponins (pavitnosides A–D (1–4))	Rhizomes of *Paris vietnamensis*	U251 and U87MG cells	100 μM	Liu et al. (2018)
Ginsenoside F2 (F2)	Intermediate metabolite formed by the hydrolysis of a glucose group on protopanaxadiol	U373MG glioblastoma cells Xenograft model using SD rat	10–60 μg/mL	Shin et al. (2012)
Polyphyllin II	*Paris polyphylla*	U251 and U87 cells	0.15625–20 μg/mL	Cheng et al. (2021)
Saponins [parpetiosides F − G (1–2)]	Rhizomes of *Paris fargesii*	U87 cells	<10 µM	Tian et al. (2024)
SB365 (saponin D)	Roots of *Pulsatilla koreana*	U87-MG, T98G cells, xenograft mice model	1–20 μM, 5 mg/kg, intratumoral	Hong et al. (2019)
CN-3	Starfish *Culcita novaeguineae*	U251 and U87 cells	0.3125–80 μg/mL	Qiu et al. (2020)

The following subsections will uncover some potent saponins that have displayed significant anti GM potential.

### 3.1 Triterpenoid saponins

Saponin B (derived from Anemone taipainsis) inhibits U87MG cell proliferation and arrests cell growth in the S phase. Moreover, saponin B-treated U87MG cells exhibited generation of apoptotic bodies and chromatin condensation at the varying doses from 1.67 to 13.35 μmol/L. The administration of Saponin B stimulated the receptor-mediated apoptotic pathway, indicating Fas-l activation. Saponin B elevated caspase and Bax expression, and diminished Bcl-2 protein expression. Therefore, saponin B significantly suppressed the growth of glioma cells by promoting apoptosis in GM cells. Therefore, it may be regarded as a viable option for the creation of innovative GM treatments with antitumor activity against gliomas (Wang et al., 2013).

Saponin 1 is a new oleanane-type triterpenoid saponin (isolated from the rhizomes of Anemone taipaiensis), is used in traditional Chinese medicine to treat phlebitis and rheumatism (Hao et al., 2017). Saponin 1 demonstrated considerable anti-cancer efficacy against human Hep-G2 cells and HL-60 cancer cells. Saponin 1 causes substantial growth suppression in both U251MG and U87MG glioma cell lines at the dose of 7.44 μg/mL. Saponin 1 induced distinct apoptotic morphological alterations in GM cells and diminished the nuclear localization and expression of NF-κB. This led to a substantial decrease in inhibitor of apoptosis proteins (IAP) expression, including that of survivin and XIAP, and the Bcl-2/Bax ratio. Saponin 1 decreased GM cell proliferation by inducing apoptosis and obstructing NF-κB-mediated cell survival. Furthermore, xenograft tumors derived from U251MG and U87MG cells exhibited suppression of tumor growth following saponin 1 administration in nude mice. In non-neoplastic astrocytes, the limited toxicity of saponin 1 indicates its substantial *in vitro* and *in vivo* anti-tumor activity, warranting further exploration as a possible treatment drug for GM (Li et al., 2013).

Phytochemical analysis of triterpenoid saponins from the roots of *Albizia lebbeck* plants resulted in the extraction of two novel oleanane-type saponins, designated lebbeckosides A and B (1–2) (Noté et al., 2015a). Comprehensive 1D and 2D NMR (13C NMR, DEPT, 1H, ROESY, TOCSY, HSQC, HMBC, and COSY) analyses, HRESIMS investigations, and chemical analyses were performed to identify their structures. The inhibitory potential of compounds 1 and 2 on the metabolism of high-grade human brain tumor cells, such as U-87 MG cell lines and TG1 cells (derived from a patient tumor), which are recognized for their notable resistance to conventional therapy, was assessed. The isolated saponins displayed significant cytotoxic activity against U-87 MG and TG1 cancer cells with IC50 values of 3.46 μM and 1.36 μM for 1, and 2.10 μM and 2.24 μM for 2, respectively (Noté et al., 2015b). Another potential madecassoside, (derived from *Centella asiatica*) exhibits several pharmacological properties. The synthesized nanoparticles were evaluated using FTIR, DSC, TGA, SEM, TEM, and DTA. Madecassoside encapsulated alginate chitosan nanoparticles (MACNP)-treated C6 glioma cells exhibit enhanced ROS generation, limited C6 proliferation, and increased uptake and distribution inside C6 cells with increasing dosage of 20, 50, 100 and 200 µg/mL. In summary, MACNP may serve as viable drug delivery vehicles for glioma therapy and provide new insights into cerebral disorders (Kunjumon et al., 2021).

Recent studies have evaluated the efficacy of Ardipusilloside I (ADS-1) (from *Ardisia pusilla*), a triterpenoid saponin, for the treatment of brain cancer. ADS-I markedly suppresses the proliferation of U373 and T98G glioma cells at the dose of 11.70 ± 0.61 μg/mL, 9.09 ± 0.22 μg/mL or 7.26 ± 0.35 μg/mL. Together with growth (cell cycle) arrest at the G2/M phase, the cytotoxic potential of ADS-I against glioma cell proliferation is linked to autophagic activation, as demonstrated by autophagosome formation and enhanced autophagic protein (Beclin1 and LC3) expression levels in glioma cells. Moreover, chloroquine, an autophagy inhibitor, diminished cell death induced by ADS-1 (Wang et al., 2014). A172, U251, and T98MG cells treated with ginsenoside Rh2 (triterpene saponin) derived from ginseng exhibited upregulation of human miRNA (miR-128) and 12 miR-128 downregulation. The pharmacological effects of P. ginseng are primarily attributed to minor ginsenosides (such as F1, F2, Rg3, Rh1, Rh2, compound Y, compound Mc, and compound K), which are generated when the sugar components of major ginsenosides are hydrolyzed. In U251 cells, transfection with an miR-128 inhibitor (50 nmol/L) inhibited Rh2 mediated miR-128 upregulation and markedly reduced caspase 3 activation, Rh2-induced cytotoxicity, E2F3a protein production, apoptosis, and E2F3a transcriptional activation (target gene of miR-128). The growth-inhibitory efficacy of ginsenoside Rh2 in human glioma cells is partially mediated by the upregulation of miRNA-128 expression reported at 10 nM (Wu et al., 2011). Targeting autophagy represents a viable therapeutic strategy in oncology (Rebecca and Amaravadi, 2016). Platycodin D (PD, from *Platycodon grandiflorus*) suppresses autophagy via upregulating the LC3B-II and p62 levels in GM cells and displayed maximum effect at a dose of 10 µM. PD inhibits the association of autophagosomes with lysosomes by promoting the storage of free cholesterol in the lysosomes, leading to its degradation. The autophagy-inhibitory role of PD was replicated via both pharmacological and genetic NPC1 suppression, which facilitated cholesterol-derived LDL export. Furthermore, PD facilitated exogenous LDL cholesterol absorption vial DL receptor activation, resulting in increased cholesterol build-up inside the lysosomes and subsequent GM cell (U87MG, U373MG) death. These phenomena were significantly more evident in LDL receptor-overexpressing GM cells than in astrocytes. Inhibition of cholesterol uptake through LDL knockdown counteracts PD-induced autophagic suppression and glioblastoma cell proliferation. Thus, PD may serve as an effective anti-GM agent by interfering with cholesterol transport and autophagy (Lee et al., 2022a).

Raddeanin A (RA), an oleanane-type triterpenoid saponin from *Anemone raddeana Regel*, inhibits the viability and spread of many malignancies (Naz et al., 2020), including glioblastoma, via diverse signaling pathways. RA (100–800 nM) was found to inhibit the proliferation, invasion and migration of GM cells via downregulation of β-catenin. It was found that RA impeded GM proliferation by suppressing invasiveness and angiogenesis in a mouse cerebral xenograft model at a dose of 100 mg/kg/day (I.P.). This study revealed that RA inhibited GM by downregulating β-catenin-mediated epithelial-mesenchymal transition and angiogenesis in GM cells and the U87 xenograft mouse model (Wu et al., 2021).

**FIGURE 2:**
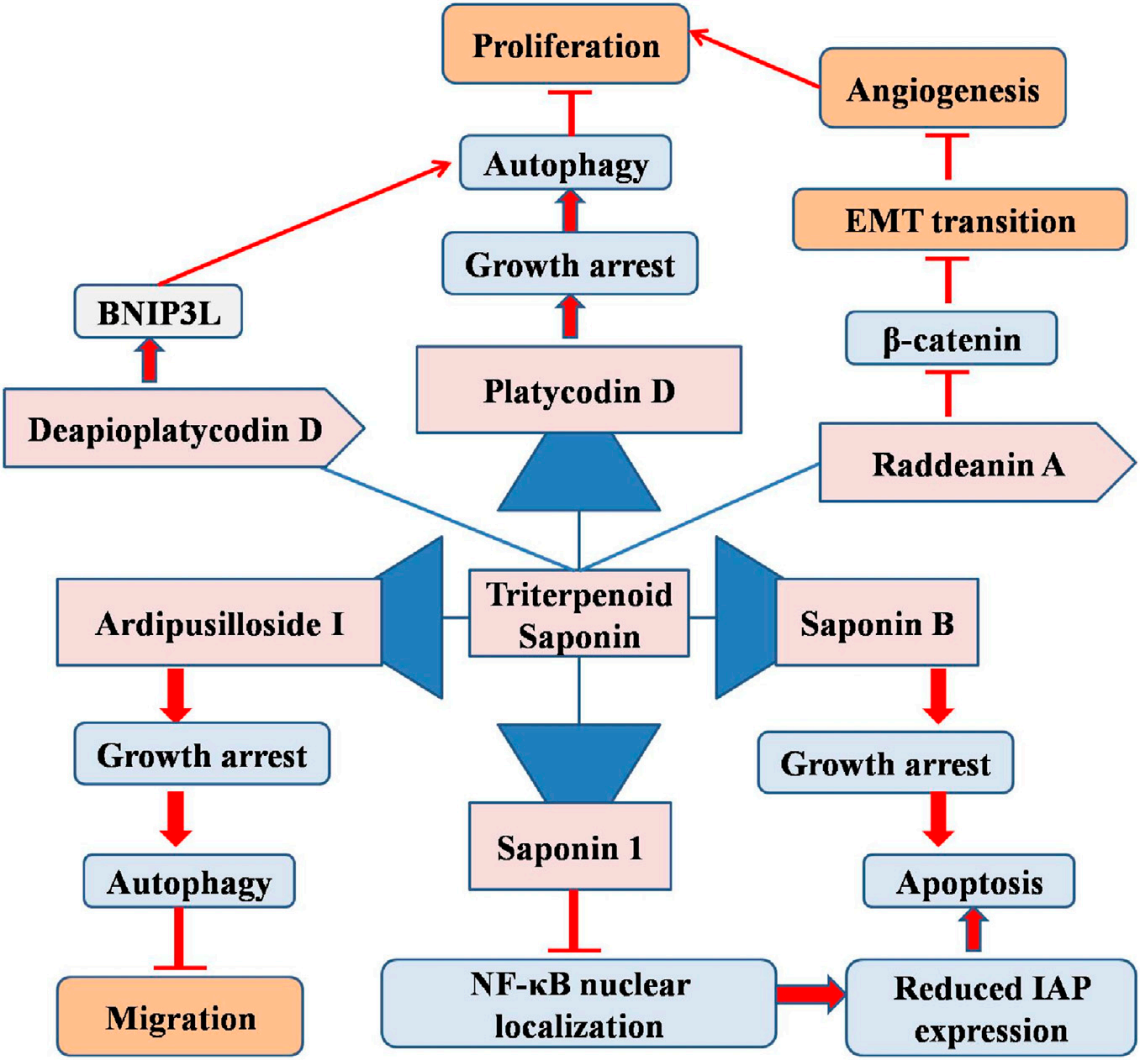
Diagram illustrating the possible mechanism associated with the anticancer potential of triterpenoid saponins against glioblastoma multiforme.

The triterpenoid saponin deapioplatycodin D (DPD) from *Platycodon grandiflorum* exhibits antiviral and anticancer effects. DPD with varying concentrations (5–80 µM) substantially suppressed the growth and development of GM cells. Under TEM, DPD-treated GM cells showed enlarged and degraded mitochondria with empty autophagic vesicles. DPD enhances autophagy in GM cells and blocks late-stage autophagic flow. Transcriptomics revealed changes in protein and mitophagy-related gene expression levels, indicating that BNIP3L enhances mitophagy in GM cells. Increased BNIP3L expression releases Beclin-1 and activates autophagy by disrupting the Bcl-2-Beclin-1 complex. After suppressing BNIP3L and overexpressing Bcl-2 in GM cells, autophagy was suppressed and DPD’s growth inhibitory effect of DPD was diminished. These results show that DPD stimulated GM cell mitophagy via BNIP3L. A mouse subcutaneous xenograft tumor model showed that DPD (4 and 8 mg/kg) through BNIP3L activates partial mitophagy in GM cells *in vivo*. Both the DPD-treated tumor model and GM cell treatment decreased GM cell growth by triggering BNIP3L-mediated partial mitophagy, thus providing a foundation for GM treatment research (Sun et al., 2025). Thus, triterpenoids could provide a novel path for the better management of glioblastoma multiforme (Figure 2).

### 3.2 Natural steroid saponin

Dioscin (a natural steroid saponin) exhibits hepatoprotective, lipid-lowering, and anti-cancer properties. Different concentrations of dioscin (1.25, 2.5, and 5 μg/mL) markedly suppressed C6 glioma cell proliferation, stimulated ROS formation, and elevated calcium ion (Ca^2+^) levels. The increase in ROS levels influences the concentrations of glutathione disulfide, malondialdehyde, glutathione, and nitric oxide, thus resulting in cellular death. Furthermore, ROS formation resulted in increased mitochondrial permeability transition and reduced MMP, which subsequently elevated (caspase 3/9) activities, facilitated cytochrome C release, and promoted the nuclear translocation of programmed cell death-5. Dioscin therapy concurrently downregulated Bcl-2/Bcl-xl protein expression while upregulating Bak, Bax, Bid, and cleaved PARP expression levels. Oxygen stress triggers S-phase arrest in cancer cells by modulating the expression of Cyclin A, DNA Topoisomerase I, CDK2, and p53, which results in DNA damage. Dioscin markedly reduced tumor size and prolonged lifespan in an allograft rat model after oral administration of 30 mg/kg of dioscin. Dioscin is a potential novel therapeutic agent for glioblastoma treatment (Lv et al., 2013).

Another steroid saponin, Paris saponin H (PSH), has also been studied in glioblastoma cells. A separate study elucidated the effectiveness of PSH in U251 glioblastoma cells and detailed the underlying mechanisms. The PSH-treated cells showed impaired cell invasion, migration, and apoptosis. PSH-treated U251 cells showed upregulation of p21 and p27 expression levels, while simultaneously downregulating cyclin D1 and Spk2 protein expression levels, inducing growth (cell cycle) arrest at the G1 phase at increasing dose from 25, 50 or 100 μg/mL. These results indicated that PSH diminished U251 cell viability by inhibiting ARA1 and ARA3 expression, further suppressing Akt and 44/42 MAPK phosphorylation, inducing apoptosis, and promoting growth (cell cycle) (Bi et al., 2021). Temozolomide (TMZ) (from Paris vietnamensis) was used to extend the overall survival of the patients. TMZ is used in Traditional Chinese Medicine and has been shown to exhibit preclinical anticancer activity in multiple cancer types. This study demonstrated that N45 (steroidal saponin) markedly inhibited glioblastoma cell proliferation, including that of U87R (TMZ-resistant glioblastoma cells), by inducing mitochondrial apoptosis via modulation of the ROS/PI3K/Akt signaling pathway. Additionally, the combination of NAC and N45 (4 μg/mL) effectively counteracted the suppression of the PI3K/Akt signaling pathway and diminished N45 induced apoptosis (Cui et al., 2024). Furthermore, N45 diminished drug resistance by downregulating NF-κB/p65, thereby reducing MGMT levels in the U87R cells. These findings demonstrate that N45 may serve as a viable therapeutic agent for glioblastoma and TMZ-resistant glioma, offering promise for mitigating drug resistance (Zhang, et al., 2020).

**FIGURE 3:**
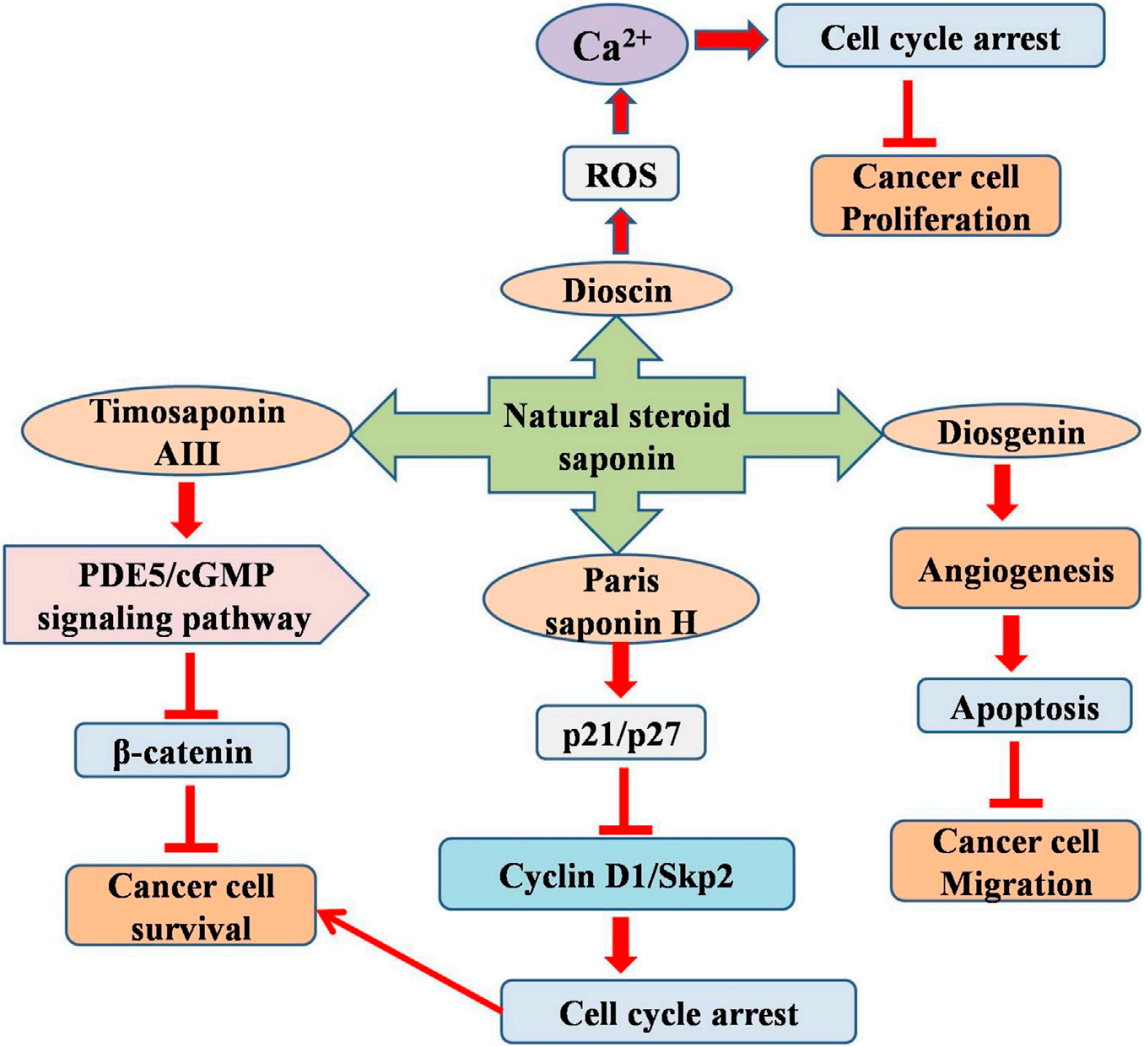
Diagram illustrating the possible mechanism associated with the anticancer potential of natural steroid saponins against glioblastoma multiforme.

Diosgenin (from fenugreek and wild yam) is a significant bioactive ingredient used to treat inflammation, diabetes, and hypercholesterolemia. Diosgenin strongly inhibits rat C6 and T98G cell proliferation Diosgenin (5–25 µM) increased the expression of the differentiation marker GFAP and reduced the expression of Id2, Notch-1, telomerase reverse transcriptase, and N-Myc in glioblastoma cells. Diosgenin inhibited migration, invasion, and angiogenesis and induced differentiation and apoptosis in glioblastoma cells (Khathayer and Ray, 2020). Timosaponin AIII (TIA), a steroidal saponin derived from *Anemarrhena asphodeloides*, has demonstrated anticancer effects across multiple cancer types (Tu et al., 2024). A study showed that TIA not only suppressed U87MG cell proliferation *in vitro* (5–10 µM) but also decreased tumor progression *in vivo* (1 mg/kg I.P.). TIA reduces the production of cGMP-specific phosphodiesterase 5 (PDE5) and increases the levels of VASPser239 phosphorylation, soluble guanylate cyclases (sGCβ), and cellular cGMP. This study posits that TIA exerts its anti-tumorigenic action by disrupting β-catenin activity via activation of the PDE5/cGMP signaling pathway, given that β-catenin is crucial for cell proliferation and survival in GM (Liao et al., 2023). Another study by Lee et al. (2022b) also demonstrated the anti-glioblastoma effects of TIA in in vitro and *in vivo* models (Lee et al., 2022a) (Figure 3).

Saikosaponin D (SSD, is a saponin derivative derived from *Bupleurum falcatum*) demonstrates an inhibitory impact on many tumor cells and is considered relatively safe at therapeutic levels (Manoharan et al., 2022). SSD (9–21 μM) markedly suppressed U251, RG-2, and U87-MG cell growth, while promoting apoptosis. Moreover, the expression of proteins associated with autophagy, the endoplasmic reticulum, and apoptosis was markedly elevated and localized in both the nucleus and cytoplasm. Consequently, SSD can be regarded as an innovative therapeutic alternative to GM. This study illustrates the anti GM effects of SSD through autophagy and cellular apoptosis (Liu et al., 2022).

The anomalous expression of MMP-9 is associated with invasion and angiogenesis of malignant tumors, along with CNS inflammation. Consequently, the synthesis of molecules capable of inhibiting or suppressing MMP-9 is essential for the treatment of brain malignancies. The ginseng saponin metabolite (compound K) markedly suppressed PMA-induced MMP-9 protein expression. K’s inhibitory effect of compound K on MMP-9 expression is associated with reduced MMP-9 promoter activity and MMP-9 mRNA levels. Moreover, compound K substantially inhibited PMA-induced activation of JNK, p38 MAPK, and ERK, which serve as AP-1 upstream regulators. Compound K suppresses the invasiveness of glioma cells *in vitro*. Consequently, the suppression of MMP-9 expression by compound K in glioma cells may provide therapeutic potential for regulating the invasiveness of brain malignancies (Jung et al., 2006).

### 3.3 Other saponins

Saponin 6 (from Anemone taipaiensis) exerts potent cytotoxic effects in HepG2 and HL-60 cells; however, anti GM efficacy remains unknown. In this study, we evaluated the effects of saponin 6 (2.83 or 5.66 µM) on U87 MG cells. Saponin 6 induces U87 MG cell death and apoptosis. This treatment also induces apoptosis, DNA fragmentation, and cell cycle arrest. Saponin 6-induced U87 MG cells apoptosis was due to increased proteins (caspase-3, -8 and -9, Fas, and Fas ligand) expression levels and reduced Bcl2 expression levels. Thus, saponin 6 has a strong therapeutic potential for GM treatment (Ji et al., 2016).

Paris saponin VII (PS VII) exhibits significant anti GM activity. PS VII impeded the migration rate of GM cells and produced growth (cell cycle) arrest at the G2/M phase, which was associated with a substantial decrease in the expression of CDK2/1 and Cyclin. Moreover, PS VII (0.25, 0.50 and 1 µM) induced cellular apoptosis by enhancing Bax expression and diminishing Bcl-2 expression, resulting in increased caspase-3 and PARP activation. PS VII efficiently inhibited PD-L1 expression via activation of the AKT1 and STAT3 signaling pathways. PS VII reinstated T cell activation in tumor/T cell co-culture by suppressing PD-L1 expression. It significantly reduced GM cell proliferation by diminishing PD-L1 levels and demonstrated that PS VII decreased volume and size, achieving superior therapeutic effects at elevated dosages of 2.5 and 5 mg/kg respectively (Wang et al., 2025).

A separate study examined the anticancer properties of the compound K (CK/ginsenoside metabolite) in GM cells. CK markedly impeded the proliferation and metastatic potential of U373MG and U87MG cells. CK inhibited growth (cell cycle) arrest at the G0/G1 phase, resulting in reduced cyclin D1/D3 expression in both the cell types. CK (50 and 75 µM) also induced death in GM cells by nuclear condensation, activated caspases (3/9) and PARP, elevated ROS production, and MMP depolarization. Moreover, CK suppressed the PI3K/Akt/mTOR signaling pathway, thereby enhancing antiproliferative and apoptotic induction. Furthermore, CK inhibited the self-renewal ability and invasiveness of U87MG and U373MG glioma stem-like cells (GSCs) by decreasing the expression of GSC markers including Sox2, CD133, Nanog, and Oct4. Collectively, these findings indicate that CK may be beneficial for GM therapy (Lee et al., 2017).

A separate study examined the anticancer efficacy of compound K (CK), a ginsenoside metabolite, in GM cells. CK markedly impeded the proliferation and metastatic potential of U373MG and U87MG cells. It further inhibited cell cycle progression in the G0/G1 phase, resulting in reduced cyclin D1 and cyclin D3 expression in both cell types. It also induces death in GM cells via nuclear condensation, elevated ROS production, MMP depolarization, and the activation of caspase-3/9 and PARP. Moreover, CK suppresses the PI3K/Akt/mTOR), thereby enhancing apoptosis. Furthermore, CK inhibits the self-renewal ability and invasiveness of U87MG and U373MG glioma stem cells by reducing the expression levels of GSC markers (Sox2, CD133, Nanog, and Oct4), suggesting that CK might be beneficial for GM therapy (Lee et al., 2022a).

Four novel spirostanol saponins (pavitnosides A–D (1–4)) along with previously identified steroidal saponins (5–10) were extracted from the rhizomes of *Paris vietnamensis*. The cytotoxicity of saponins was assessed in U251 and U87MG cells. It further demonstrated minimal anti-proliferative action against U87MG cells, whereas established saponins 8/9 showed considerable cytotoxicity against both tumor cell lines (Liu et al., 2018). Notwithstanding the combination of radiation and chemotherapy, survival durations remain exceedingly short. Ginsenoside F2 is an intermediate metabolite, formed by the hydrolysis of a glucose group on protopanaxadiol (PPD), and is subsequently converted into either Rh2 or compound K. Consequently, an additional study evaluated the efficacy of ginsenoside F2 (F2) in combating glioblastoma cells U373MG. F2 (10–60 μg/mL) exhibited cytotoxicity and induced cell cycle arrest at the sub-G1 phase, indicating apoptosis (Shin et al., 2012). In a xenograft model using SD rats, F2 diminished tumor development and showed anticancer efficacy by inhibiting Ki67 proliferation and inducing death via caspase3/8 activation. Reduced expression of CD31 indicates a decrease in blood vessel density. The expression which matrix metalloproteinase-9 associated with cancer invasion was also suppressed. The number of cells expressing the cell markers CD133 and nestin was reduced. The results of this study indicated that F2 may serve as a novel chemotherapeutic agent for GB treatment by impeding cancer development and invasion (Shin et al., 2012).

Polyphyllin II (from *Paris polyphylla*) with varying concentrations (0.15625–20 μg/mL) exhibited significant suppression to growth, invasion and induce mitochondrial apoptosis in both U251 and U87 cells. Polyphyllin II promotes Bax, cleaved-caspase 3, p-AKT, cyt-c, and caspase 3 and reduces Bcl-2and AKT (Cheng et al., 2021). Two previously unexplored cholestanol saponins [parpetiosides F − G (1–2)] and six known analogs (3–8) have been isolated (from the rhizomes of *Paris fargesii*. The cytotoxicity of saponins (1–8) against three human cancer cell lines (SGC-7901, U87, and HepG2) and saponins 5–8 displayed certain cytotoxicity (Tian et al., 2024).

### 3.4 Green tea seed isolated saponin

Asaponin E1 (from the ethanol extract of green tea seeds) displayed inhibitory effect on tau hyperphosphorylation and neuroinflammation in neuroblastoma (SHY-5Y) and glioblastoma (HTB2) cells. Theasaponin E1 substantially and dose-dependently reduced tau hyperphosphorylation by inhibiting the expression levels of PICALM, CAMII, CDK5, MAPK, GSK3 β, and EPOE4 (E4), while augmenting the expression levels of Aβ, TREM2, p-tau, PP1, APP, and PP2A, Furthermore, theasaponinE1 mitigated inflammation by inhibiting the NF-kB pathway and reducing the levels of inflammatory cytokines, including IL (beta, 6) and TNF-alpha (Khan et al., 2022). These could be utilized as potent target for asaponin for elucidating better therapeutic option. To identify novel saponins, phytochemical analysis of the roots of *Albizia boromoensis* resulted in the isolation of four new boromoenosides A–D (1–4) (olenane-type saponins). Data analysis of these saponins was performed using direct analysis of spectral data, primarily 1D NMR, HRESIMS, and 2D NMR, along with comparison with existing research data. None of the extracted saponins showed cytotoxicity against U-87 MG human glioma cell lines or TG1 glioblastoma stem-like cells (Note et al., 2015b). SB365 (from the roots of Pulsatilla koreana) has been documented to exhibits cytotoxicity in numerous cancer cells. Nonetheless, the efficacy of SB365 in U87-MG and T98G glioma cells, as well as its usefulness in conjunction with temozolomide for the treatment of glioma, is under investigation.

SB365 (saponin D) exerts a cytotoxic effect on GM by inducing apoptosis and initiating caspase-independent cell death. Inhibition of autophagic flow and neutralization of lysosomal pH occurred swiftly after the administration of SB365 (0–20 µM), subsequently leading to a decline in mitochondrial membrane potential. SB365, in conjunction with temozolomide, demonstrated additive cytotoxic effects both *in vitro* and *in vivo*. In summary, SB365 inhibits autophagic flow and triggers caspase-independent cell death in GM cells via mechanisms involving cathepsin B and mostly reactive oxygen species, and its combination with temozolomide demonstrates potential for the treatment of GM (Hong et al., 2019). CN-3 (extracted from the starfish *Culcita novaeguineae*) inhibits the growth of U251 and U87 GM cells at low doses. A microarray study showed that CN-3 (0, 0.3125, 0.625, 1.25, 2.5, 5, 10, 20, 40, and 80 μg/mL) significantly enhanced the differential expression of 661 genes associated with anti-glioma activity in U251 cells. CN-3 mediated downregulated of SCUBE3 expression facilitates both growth (cell cycle) G1/S arrest and inhibition of U251 via the p27/E2F1/p-Akt/p53/p21 signaling pathway (Qiu et al., 2020).

## 4 Conclusion and future perspective

Saponins exhibits a variety of biological functions, such as cytotoxic, antiproliferative, and pro-apoptotic effects on cancer cells, making them effective treatment agents for GM. Their ability to traverse the blood-brain barrier, modulate signaling pathways, and induce oxidative stress in tumor cells makes them promising candidates for novel or supplementary treatment options for GM. Their membrane-active properties and amphiphilic structure may allow partial permeability across the blood–brain barrier (BBB), which would be advantageous for treating brain malignancies where medication delivery remains a major challenge. In addition, the structural diversity and natural origin of these molecules present prospects for the development of more specific and less toxic anticancer agents. Altogether, raddeanin A, acacic acid-type saponins, paris saponins are some of the reported natural saponins that have shown strong anti-glioblastoma effects at lower doses in different cell lines in comparison to other compounds. On the other hand, timosaponin A III, saponin A, and deapioplatycodin D showed significant antitumor potential at effective dose ranges in GM animal models. This suggests that these compounds could be considered for further preclinical and clinical research for the management of GM.

Despite several benefits of saponins, and promising potential in preclinical models, considerable obstacles persist in the clinical implementation of GM therapy. Saponins often exhibit poor bioavailability, limited stability, and dose-dependent toxicity, potentially constraining their therapeutic effects. The complex structure of the blood-brain barrier further exacerbates concerns about the efficient delivery of natural compounds like saponins to the central nervous system and their pharmacokinetics in humans. One of the biggest challenges in developing CNS therapeutic agents is the BBB’s impermeability to most therapeutics. To treat GM, it is imperative to overcome the BBB’s blocking function, enhance drug accumulation at tumor sites, and reduce harmful side effects. In order to overcome these challenges, researchers have developed a range of innovative materials and technologies to enhance the delivery of brain-targeted drugs by carefully and non-invasively modulating the BBB. Among these strategies, targeted drug delivery systems have shown considerable promise.

Targeted drug delivery systems provide several benefits for the therapeutic use of natural compounds such as saponins. These technologies increase therapeutic concentration at the target site while reducing systemic toxicity and off-targets effects by precisely targeting natural compounds to the damages tissues or cells. This targeted strategy increases therapeutic efficacy, reduces the required dosage, and helps overcome issues such as poor solubility, low bioavailability, and rapid metabolism of natural compounds. Furthermore, unstable phytochemicals can be protected from degradation and released in a regulated manner using ligand-based targeting and nanocarriers. Taken together, targeted drug delivery systems offer a viable approach to optimize the clinical. Potential of saponins and promote their transformation into contemporary, safe, and effective treatments. Despite their enormous potential, these targeted approaches for natural compounds encounter multiple challenges and constraints. The intricate structure and variable compositions of these compounds often complicate large-scale manufacturing, formulation stability, and reproducibility. Numerous targeting strategies require complex nanocarriers, or surface modifications, which can increase production costs and pose challenges for regulatory approval, long term safety, and biocompatibility. Ultimately, although saponins showed great promise as anticancer agents against GM. Further preclinical research and well-designed clinical trials are necessary to completely evaluate their viability, safety and effectiveness in clinical oncology.
